# Kefir-fermented soymilk reduces exercise-induced fatigue in mice by influencing the gut microbiota and short-chain fatty acid metabolism

**DOI:** 10.3389/fmicb.2026.1881421

**Published:** 2026-06-23

**Authors:** Xiaoyu Wang, Junshun Zhang, Jiayi Ge, Danning Wu, Mengyuan Shao, Xiaochen Huang, Zhina Chen

**Affiliations:** 1 School of Physical Education, Huainan Normal University, Huainan, China; 2 School of Biological Engineering, Huainan Normal University, Huainan, China; 3 School of Food and Pharmaceutical Engineering, Zhaoqing University, Zhaoqing, China

**Keywords:** exercise-induced fatigue, fermented soymilk, gut microbiota, kefir, short-chain fatty acids

## Abstract

Fermented foods have obtained increasing attention because of their potential health advantages, particularly in modulating gut microbiota and metabolic functions. However, the impacts of fermented soymilk on exercise-induced fatigue and its basic mechanisms remain unclear. Here, mice were gavaged fermented soymilk (FM), unfermented soymilk (BM), or normal saline (Control) for 30 days, followed by an exhaustive swimming test. Fatigue-related biochemical parameters, antioxidant indices, gut microbiota composition, fecal short-chain fatty acids (SCFAs), and KEGG functional pathways were analyzed. FM significantly extended exhaustive swimming time compared with the Control and BM groups. It reduced serum LDH and BUN levels, increased glycogen storage, and improved antioxidant capacity, as indicated by elevated CAT activity and reduced MDA levels. FM markedly reshaped the gut microbiota by enriching SCFA-producing genera, including *Blautia*, *Faecalibacterium*, *Dysosmobacter*, *Roseburia*, and *Lachnoclostridium*, while reducing opportunistic pathogens. It enhanced carbohydrate and amino acid metabolism, as well as microbial interaction pathways, thereby promoting the synthesis of acetate and butyrate. These findings suggest that FM improves exercise performance and alleviates fatigue by enhancing energy metabolism, reducing oxidative stress, and modulating gut microbiota and its metabolic functions.

## 1 Introduction

Physical fatigue is a common physiological phenomenon that happens after prolonged or vigorous physical activity and is characterized by decreased physical performance and reduced work capacity (Fang et al., 2022; Li S. et al., 2025). The development of fatigue involves multiple physiological factors, including energy depletion, accumulation of metabolic by-products, oxidative stress, and muscle damage (Cai et al., 2010). During intense exercise, glycogen stored in the liver and skeletal muscles serves as the primary energy source (Hearris et al., 2018). Overconsumption of glycogen reserves and deposition of metabolic products, including lactate and blood urea nitrogen (BUN), impair muscle function and contribute to fatigue development (Zhao Z. et al., 2026). In addition, strenuous exercise increases production of reactive oxygen species (ROS), leading to oxidative stress and cellular structure damage (Carey et al., 2021). Therefore, enhancing energy metabolism, attenuating oxidative stress, and alleviating metabolic accumulation are key strategies for preventing exercise-induced fatigue (Zhou et al., 2023).

In recent years, natural antifatigue agents derived from functional foods have attracted increasing attention. Compared with synthetic supplements, food-derived bioactive compounds are generally considered safer and more suitable for long-term consumption. Various natural products, including plant extracts (He et al., 2022; Jia and Zhao, 2022), polysaccharides (Jiang et al., 2023; Jing et al., 2023), peptides (Cai et al., 2023; Feng et al., 2021; Liu et al., 2020, 2023), and fermented foods, have been shown to alleviate fatigue through multiple mechanisms, such as enhancing glycogen storage, improving antioxidant capacity, and regulating metabolic pathways.

Soymilk, a soybean-derived beverage, is widely consumed for its nutritional value and has been reported to provide multiple health benefits, including reducing cholesterol, lowering lipids, antioxidant, anti-inflammatory, anticancer, and metabolic regulatory effects (Nande et al., 2008; Jayachandran and Xu, 2019). However, the bioavailability of various nutrients in soymilk may be limited by the presence of antinutritional factors. Fermentation serves as an efficient strategy for enhancing both the nutritional value and functional characteristics of soybean-derived products (Lee et al., 2023). Microbial fermentation can degrade antinutritional compounds, produce bioactive peptides, and enhance the bioavailability of nutrients, thereby increasing the biological activity of fermented foods.

Kefir, a traditional fermented food, is produced by the action of a complex microbial consortium composed of lactic acid bacteria (LAB), yeasts, and acetic acid bacteria (AAB) (Garofalo et al., 2020). Kefir has been widely studied for its probiotic and functional properties. Kefir and kefir-derived fermented products exert beneficial effects, including antioxidant activity, immune regulation, antimicrobial effects, and metabolic improvement (Saleem et al., 2023). When kefir is used for soymilk fermentation, various bioactive compounds can also be produced, thereby enhancing the physiological functions of fermented soymilk (Luo J. et al., 2023; Egea et al., 2023).

In addition to direct metabolic effects, recent investigations have emphasized the important role of gut microbiota in regulating host metabolism and physical performance (Czarnota et al., 2025). The gut microbiota forms a complex ecosystem that participates in nutrient metabolism, immune regulation, and production of bioactive metabolites (Sommer and Bäckhed, 2013). Accumulating evidence suggests that alterations in gut microbial composition can influence energy utilization, inflammatory responses, and oxidative stress, all of which are closely linked to fatigue development (Czarnota et al., 2025; Li Z. et al., 2025). Therefore, modulating the gut microbiota has been suggested as a potential approach to improve exercise endurance and reduce fatigue (Zhao Z. et al., 2026). One of the most important functional metabolites produced by gut microbiota is short-chain fatty acids (SCFAs), primarily including acetate, propionate, and butyrate (Liu et al., 2025). These metabolites are generated by bacterial anaerobic fermentation of dietary components and serve as important signaling molecules and energy substrates for the host (Frampton et al., 2020). SCFAs regulate glucose and lipid metabolism, enhance mitochondrial function, and reduce inflammation (Martin-Gallausiaux et al., 2021). Furthermore, SCFAs may contribute to improved exercise performance by providing additional energy sources and modulating metabolic pathways associated with endurance capacity (Liu et al., 2025; Mach and Fuster-Botella, 2017).

Despite increasing research on functional fermented foods, limited information is available regarding the antifatigue effects of kefir-fermented soymilk and its potential mechanisms involving gut microbiota and microbial metabolites. For this purpose, the antifatigue properties of kefir-fermented soymilk were evaluated in a mouse model using a weight-loaded swimming test. Biochemical indicators related to fatigue, glycogen metabolism, oxidative stress, gut microbiota composition, and fecal SCFAs were analyzed to explore the underlying mechanisms. The findings may provide new understanding of the functional properties of kefir-fermented plant-based beverages and contribute to the development of novel functional foods for improving physical endurance and alleviating exercise-induced fatigue.

## 2 Materials and methods

### 2.1 Materials

Kefir grains utilized in the present research were preserved in the Food Microbiology Laboratory of Huainan Normal University.

### 2.2 Sample preparation

A total of 400 g of soybeans with plump grains, free from mold and insect damage, were weighed and soaked in water at a 1: 3 (w/v) ratio for 8 h. After soaking, the beans were ground with a grinder at a 1: 7 (w/v) soybean-to-water ratio. Subsequently, each 250 mL Erlenmeyer flask received 100 mL of the prepared soymilk and was then treated with sterilization at 121 °C for 20 min (Chen et al., 2025). After cooling to room temperature, activated kefir grains were added to 100 mL of soymilk at an inoculation concentration of 5% (w/v). Then the sample was incubated in a biochemical incubator at 25 °C for 24 h. The fermented soymilk was centrifuged at 7,000 rpm for 20 min, and the supernatant was filtered to remove solids, then frozen at −20 °C. The frozen supernatant of fermented soymilk and unfermented soymilk were separately freeze-dried to powder and dissolved to 11 mg/mL for gavage.

### 2.3 Animal experiment

Male BALB/c mice, aged 7 weeks and of SPF grade, were bought from Henan Skebes Biotechnology Co., Ltd. All animal experiments were approved by the Experimental Center Ethics Committee of Huainan Normal University (Approval No. HNNU-20251118). The animals were reared in a controlled environment featuring a relative humidity of 45%–55%, an ambient temperature of 22 °C ± 1 °C, and a 12-h light/dark cycle (Lu et al., 2023). After a 7-days adaptation, mice were randomly allocated into three groups (*n* = 10 per group): normal saline (Control), unfermented soymilk (50 mg/kg body weight, BM), fermented soymilk group (50 mg/kg body weight, FM). The oral gavage was carried out at the same time daily for a total of 30 days. The weight of mice was measured and recorded every day. Thirty minutes after the last gavage, each mouse was subjected to a weight-loaded swimming test. A lead wire was wrapped around the tail to provide an additional load of 5% of the body weight, with the tightness adjusted appropriately. The mice were then placed into a swimming tank filled with water to a depth of 30 cm, with the water temperature maintained at 25 °C ± 1 °C. The timer was started upon entry into the water, and the mouse was kept actively swimming throughout. Exhaustion was defined as the inability to resurface after the head had remained submerged for more than 7 s, at which point the mouse was immediately removed, and the exhaustive swimming time was recorded (Cai et al., 2023; Lu et al., 2023).

### 2.4 Measurement of fatigue-related biochemical parameters

Following the swimming test, blood samples were obtained, and blood levels of blood lactic acid (BLA), BUN, lactate dehydrogenase (LDH), creatine kinase (CK), catalase (CAT), and malondialdehyde (MDA) were measured using assay kits provided by Nanjing Jiancheng Biotechnology Co., Ltd., Jiangsu, China (Cai et al., 2023).

After blood collection, the mice were necropsied. Organ index (heart, liver, spleen, and kidneys) were calculated according to the individual body weight. Liver and muscle glycogen contents were then detected using assay kits provided by Nanjing Jiancheng Biotechnology Co., Ltd., Jiangsu, China (Lu et al., 2023).

### 2.5 Gut microbiota analysis

For each group, five fresh fecal samples from mice were subjected to metagenomic sequencing. Total microbial DNA was extracted by TIANamp Soil DNA Kit DP336. Agilent 5400 was used to determine the quality of DNA extraction. After constructing the library, the samples were subjected to metagenomic sequencing using Rapid Plus DNA Lib Prep Kit for Illumina (Li et al., 2023). Kraken2 was used to classify sequencing reads against a microbial database, and relative abundance of species was then estimated by Bracken (Wu et al., 2026). After aligning the quality-controlled and host-depleted sequences against the protein database, relative abundance tables and functional annotations for each functional database were obtained. Based on the taxonomic abundance matrix and functional abundance table, abundance-based cluster analysis and principal coordinate analysis (PCoA) were performed. Furthermore, LEfSe analysis was conducted to identify biomarkers that distinguish species and functional composition among the samples.

### 2.6 Determination of SCFAs in feces

Fecal SCFAs concentrations were detected by gas chromatography-quadrupole mass spectrometry according to established methods (Cui et al., 2024; Zhao J. et al., 2026). Briefly, obtain an appropriate volume of fecal sample and add it to a centrifuge tube. Then, add 50 μL of 30% phosphoric acid solution and 300 μL of acetone solution. Mix thoroughly for 3 min, and then centrifuge at 12,000 rpm for 10 min. The supernatant was diluted according to the actual situation before being tested. The Agilent 7820 gas chromatography equipped with a DB-FFAP column was used to quantify. The Agilent 5977 quadrupole mass spectrometry detection equipped with an electron impact ion source (EI) was used to detect and quantify the SCFAs in fecal samples.

### 2.7 Statistical analysis

All statistical analyses in this study were carried out using SPSS v22.0. Differences between groups were assessed by one-way ANOVA. The experimental results were obtained from at least three independent replicates, and the data were shown as mean ± standard deviation (SD). A *p*-value of less than 0.05 was considered statistically significant.

## 3 Results

### 3.1 Effects of fermented soymilk on exercise-induced fatigue in mice

**FIGURE 1:**
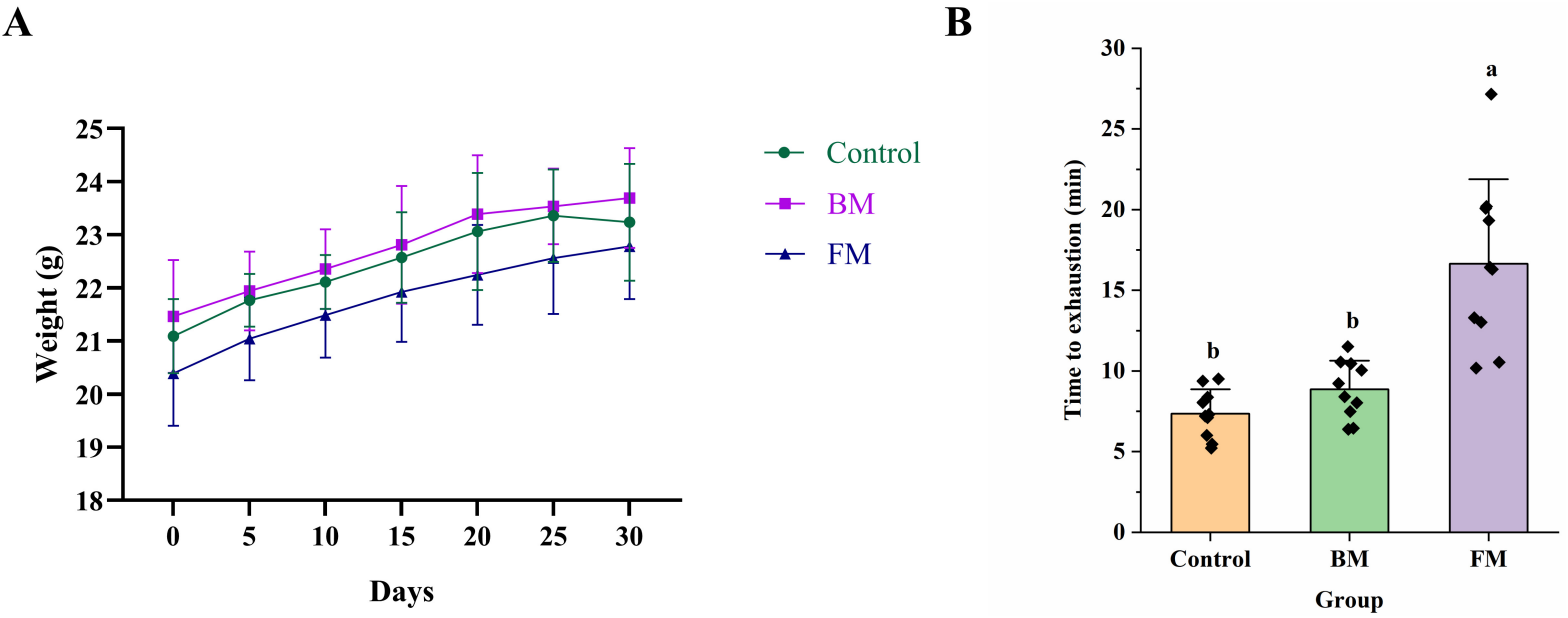
The antifatigue properties of fermented soymilk on BALB/c mice. **(A)** Body weight gain. **(B)** Time to exhaustion in exhaustive swimming test. Control: normal saline, BM: unfermented soymilk, FM: fermented soymilk. Different lowercase letters indicate significant difference at *p* < 0.05.

During the trial, all groups exhibited a steady increase in body weight, with no statistically significant differences observed (Figure 1A), indicating that the fermented and unfermented soymilk had no impact on normal growth. No mortality or abnormal behaviors were observed throughout the experiment, and all mice exhibited normal food and water intake, and glossy hair, suggesting that fermented and unfermented soymilk had no observable toxicity. Moreover, organ indices did not differ significantly among groups (Table 1), suggesting no adverse effects on major organs.

**TABLE 1:** Effect of fermented soymilk on organ indices of mice.

Group	Cardiac index (%)	Liver index (%)	Spleen index (%)	Kidney index (%)
Control	0.63 ± 0.01^a^	4.24 ± 0.27^a^	0.41 ± 0.03^a^	1.47 ± 0.13^a^
BM	0.62 ± 0.02^a^	4.28 ± 0.53^a^	0.42 ± 0.06^a^	1.46 ± 0.09^a^
FM	0.62 ± 0.07^a^	4.38 ± 0.29^a^	0.46 ± 0.11^a^	1.44 ± 0.13^a^

Control: normal saline, BM: unfermented soymilk, FM: fermented soymilk. Different superscript letters indicate significant differences among groups (*p* < 0.05).

Exercise endurance was assessed using the exhaustive swimming test. As shown in Figure 1B, the exhaustion swimming times for the Control, BM, and FM groups were 7.36 ± 1.50 min, 7.14 ± 1.57 min, and 16.65 ± 5.23 min, respectively. The FM group had a significantly longer swimming time than both the Control and BM groups (*p* < 0.05), whereas no statistically significant difference was found between the Control and BM groups (*p* > 0.05). These results showed that fermented soymilk supplementation significantly enhanced exercise endurance, while unfermented soymilk showed no evident effect.

### 3.2 Effects of fermented soymilk on fatigue-associated biochemical parameters

**FIGURE 2:**
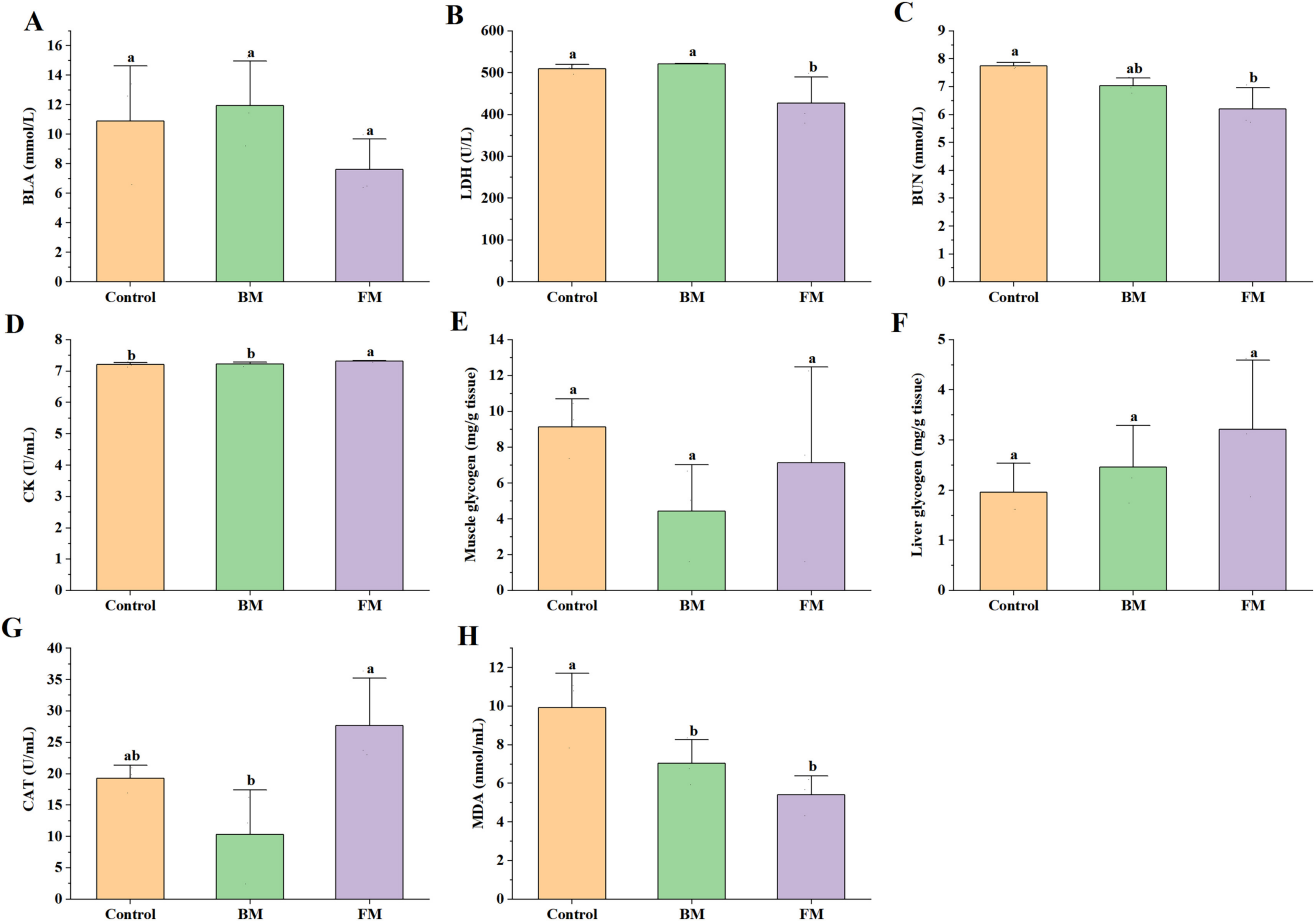
Effects of fermented soymilk supplementation on biochemical indices related to the antifatigue in mice. **(A)** BLA, **(B)** LDH, **(C)** BUN, **(D)** CK, **(E)** muscle glycogen, **(F)** liver glycogen, **(G)** CAT, and **(H)** MDA. Control: normal saline, BM: unfermented soymilk, FM: fermented soymilk. Different lowercase letters indicate significant difference at *p* < 0.05.

Lactate is a metabolic byproduct produced during anaerobic exercise, and elevated lactate levels can lead to muscle fatigue. The BLA concentrations of three groups of mice were 10.88 ± 3.74 mmol/L, 11.95 ± 3.02 mmol/L, and 7.63 ± 2.03 mmol/L, respectively. Although the FM group showed a lower BLA concentration than the Control and BM groups, no statistical significance was observed for this reduction (Figure 2A, *p* > 0.05). LDH, a marker of muscle damage, was significantly reduced in the FM group relative to the others (Figure 2B, *p* < 0.05), indicating reduced muscle injury. BUN reflects protein metabolism status. Compared with the Control group, the FM group showed a significantly lower BUN level (Figure 2C, *p* < 0.05), suggesting that fermented soymilk could help reduce protein breakdown during exercise, protecting muscle proteins from excessive depletion. However, no significant differences in LDH or BUN levels were found between the BM group and the Control group.

CK is a key indicator of muscle injury after intense exercise. Normally low in serum, CK rises when strenuous exercise damages muscle cells and increases membrane permeability, allowing CK to leak into the bloodstream. Therefore, elevated serum CK levels indicate muscle damage. As depicted in Figure 2D, serum CK levels were 7.23 ± 0.06 U/mL (Control), 7.24 ± 0.04 U/mL (BM), and 7.31 ± 0.02 U/mL (FM), with no significant differences among groups (*p* > 0.05). These results indicated that exercise-induced muscle damage was not severe under the experimental conditions.

Glycogen is a major energy source during exercise. Figures 2E,F showed that the FM group had higher muscle and liver glycogen levels than both the Control and BM groups. This suggested that fermented soymilk may increase available energy stores during exercise by promoting glycogen synthesis or reducing glycogen consumption, thereby delaying fatigue. In addition, oxidative stress markers revealed that CAT activity was significantly increased, while MDA levels were significantly decreased in the FM group (Figures 2G,H). In addition, compared with the Control group, oxidative stress markers revealed significantly increased CAT activity and decreased MDA levels in the FM group, whereas no significant differences were found in the BM group. These results indicated that fermented soymilk improved antioxidant capacity and reduced exercise-induced oxidative damage.

### 3.3 Regulation effect of fermented soymilk on gut microbiota in mice

**FIGURE 3:**
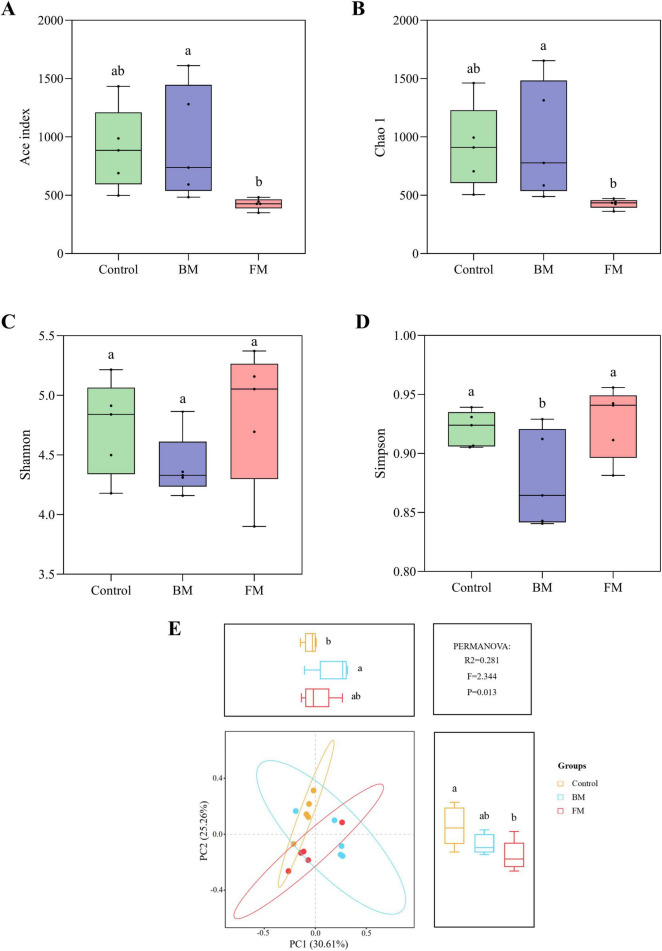
Impact of fermented soymilk on the abundance and diversity of gut microbiota. **(A)** ACE index, **(B)** Chao1 index, **(C)** Simpson index, **(D)** Shannon index. **(E)** Beta diversity. Control: normal saline, BM: unfermented soymilk, FM: fermented soymilk. Different lowercase letters indicate significant difference at *p* < 0.05.

Alpha diversity analysis (Figures 3A–D) revealed that fermented soymilk did not significantly alter the Ace or Chao1 indices in the FM group compared with the Control group (*p* > 0.05), suggesting that total species richness was not increased. However, the Shannon index was elevated and the Simpson index was reduced in the FM group, indicating an improvement in microbial evenness and a reduction in the dominance of a few taxa. In contrast, the BM group showed a significant decrease in the Simpson index relative to the Control group (*p* < 0.05), indicating a reduction in the dominance of certain taxa, although no significant changes were observed in the Ace, Chao1, or Shannon indices, suggesting that unfermented soymilk partially affected community evenness without altering overall species richness or overall diversity.

Beta diversity assesses compositional differences in microbial communities among groups. As shown in Figure 3E, PCoA based on operational taxonomic unit (OTU) levels showed a clear separation among the three groups (*p* = 0.013). Along the PC1 and PC2 axes, the FM group was significantly separated from the Control group, whereas the Control and BM groups were not significantly separated. These results indicated that fermented soymilk markedly altered the gut microbiota structure.

**FIGURE 4:**
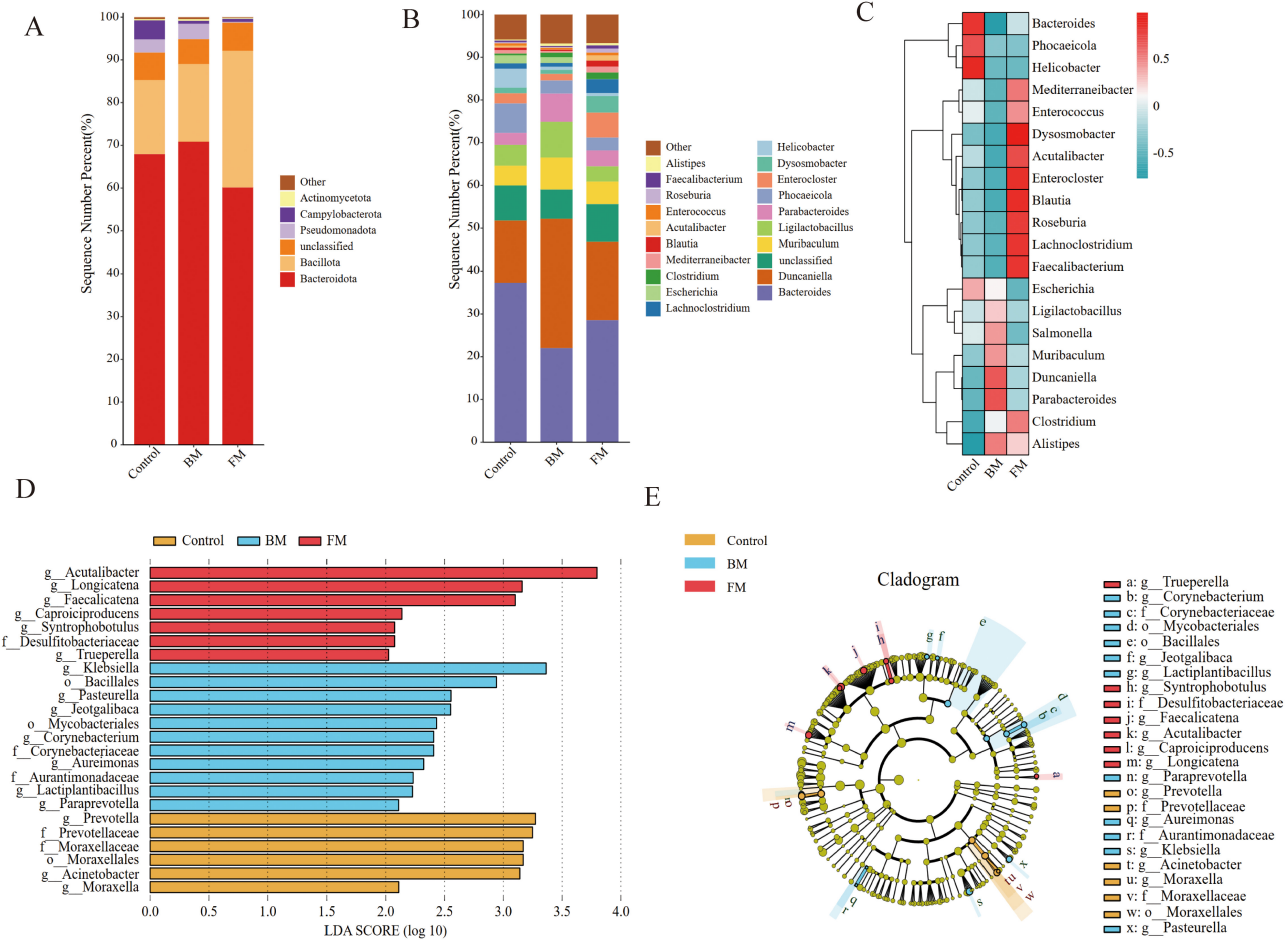
(A) Phylum-level relative abundance of the microbial community, **(B)** Genus-level relative abundance of the microbial community, **(C)** Heatmap of the differential microorganisms at the genus among all groups, **(D)** Distribution histogram based on linear discriminant analysis LDA (LDA score > 2.0, *p* < 0.05), **(E)** Cladogram representation of taxa using linear discriminant analysis effect size. Control: normal saline, BM: unfermented soymilk, FM: fermented soymilk.

At the phylum level, Bacteroidota and Baciilota dominated across all groups (Figure 4A), accounting for 80%–90% of the total samples. In addition, the relatively abundant phylum also included unclassified, Actinomycetota, Campylobacterota, and Pseudomonadota. Relative to the Control group, the FM group exhibited a notable increase in the abundance of Bacillota in the cecal contents, the content of Bacteroidota, Pseudomonadota, Campylobacterota, and Actinomycetota decreased. In contrast, the BM group showed an increase in the abundance of Bacteroidota and Pseudomonadota.

Furthermore, at the genus level, the predominant members of the gut microbiota in all groups, including *Bacteroides*, *Duncaniella*, *Muribaculum*, *Ligilactobacillus*, and *Parabacteroides* (Figure 4B). Relative to the Control group, the BM group exhibited a significantly higher relative abundance of *Duncaniella*, *Ligilactobacillus*, *Muribaculum*, and *Parabacteroides*, which increased from 14.5%, 4.9%, 4.6% and 2.8% to 30.2%, 8.4%, 7.5% and 6.6%, respectively. In comparison with both the Control and BM groups, the FM group exhibited more pronounced gut microbiota remodeling, characterized by the enrichment of *Enterocloster*, *Dysosmobacter*, *Lachnoclostridium*, *Mediterraneibacter*, *Blautia*, *Acutalibacter*, *Roseburia*, and *Faecalibacterium* (Figure 4C). These genera all belong to the phylum Bacillota, and predominantly typical anaerobic fermentative bacteria that can utilize dietary substrates to produce SCFAs, particularly butyrate and acetate, thereby contributing to the maintenance of intestinal barrier function and the regulation of host metabolism. *Roseburia* and *Faecalibacterium* are regarded as important marker genera of gut health, and increases in their abundance are generally associated with an anti-inflammatory state and improved metabolic function. Meanwhile, the relative abundances of the opportunistic pathogenic genera *Helicobacter* and *Escherichia* were significantly decreased in the FM group, from 4.4% and 1.9% to 0.7% and 0.03%, respectively. Linear discriminant analysis effect size (LEfSe) and cladogram analysis further confirmed the enrichment of putative SCFA-producing taxa, including *Faecalibaculum* and *Acutalibacter*, in the FM group (Figures 4D,E). These findings indicated that fermented soymilk exerted a stronger regulatory effect on the gut microbiota than unfermented soymilk, promoting a more beneficial microbial profile.

### 3.4 Effects of fermented soymilk on SCFAs levels in mice feces

**FIGURE 5:**
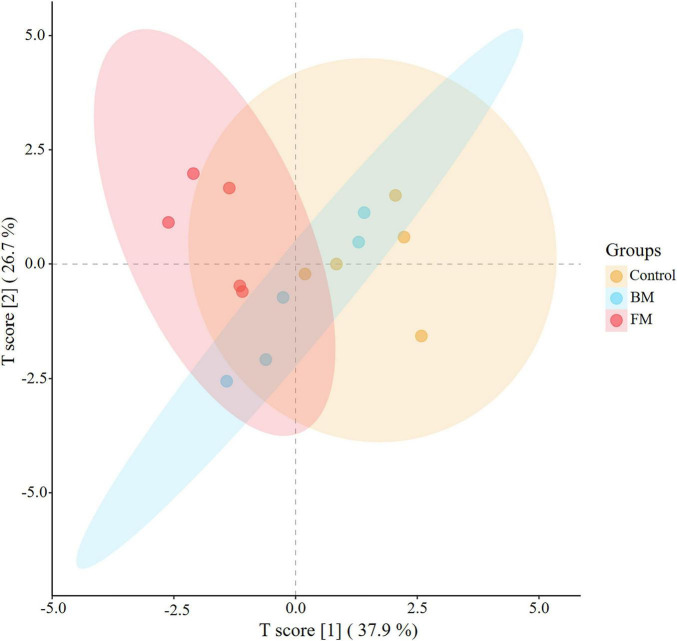
Partial least squares discriminant analysis (PLS-DA, VIP > 1.5, FDR < 0.05) of SCFAs in the fecal contents. Control: normal saline, BM: unfermented soymilk, FM: fermented soymilk.

**FIGURE 6:**
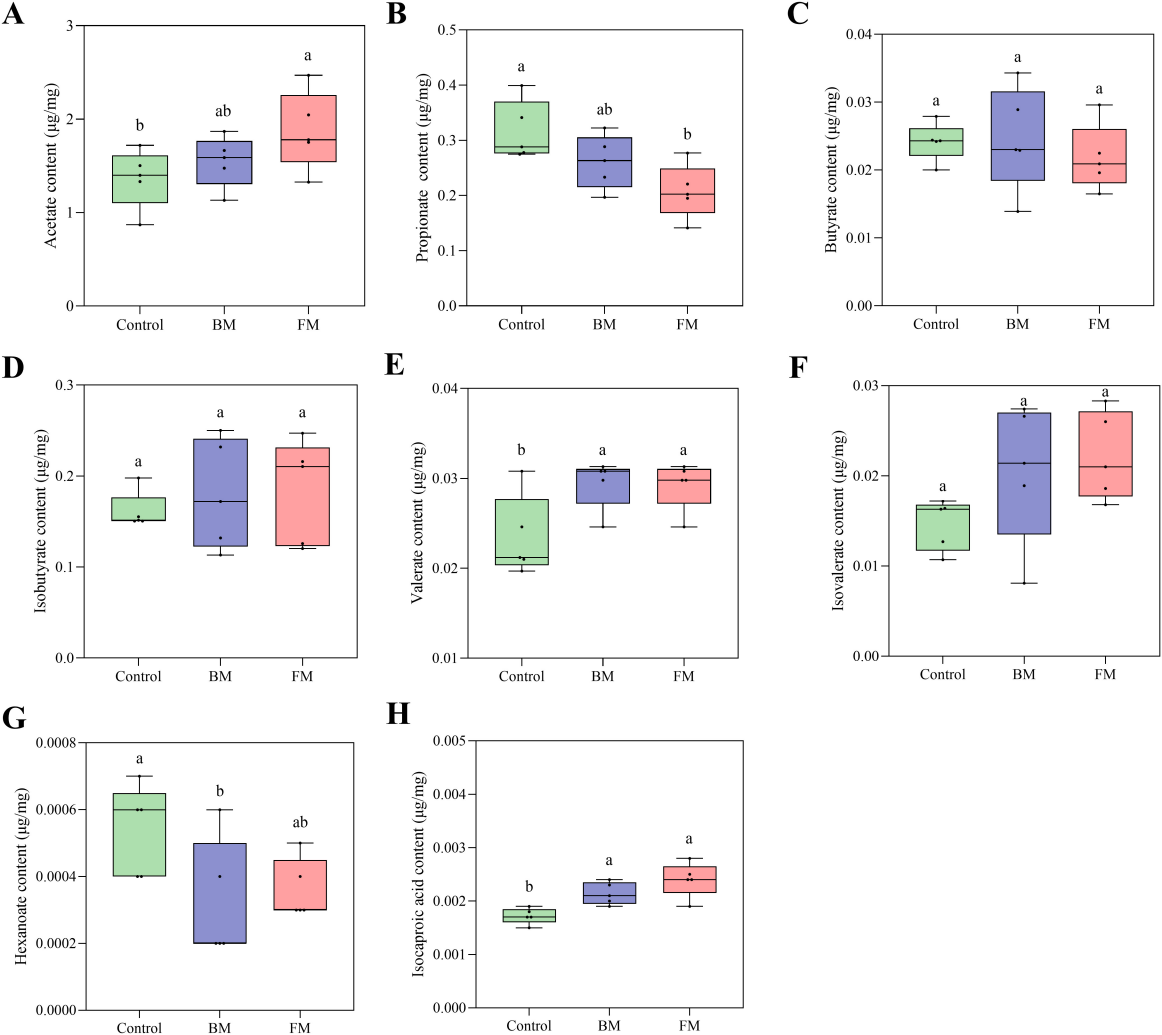
Concentrations of eight SCFAs in mouse feces. **(A)** Acetate, **(B)** Propionate, **(C)** Butyrate, **(D)** Isobutyrate, **(E)** Valerate, **(F)** Isovalerate, **(G)** Hexanoate, **(H)** Isocaproic acid. Control: normal saline, BM: unfermented soymilk, FM: fermented soymilk. Different lowercase letters indicate significant difference at *p* < 0.05.

The detection results of fecal SCFAs are presented in Figures 5, 6. PLS-DA analysis revealed a clear separation of the FM group from both the Control and BM groups (Figure 5), indicating that fermented soymilk induced a systemic alteration in the SCFA metabolic profile of mice, whereas unfermented soymilk had no obvious effect. Quantitative analysis showed that acetate, propionate, butyrate, isobutyrate, valerate, and isovalerate were the predominant SCFAs. Compared to the Control group, the acetate concentration in the FM group increased significantly from 1.36 ± 0.31 μg/mg to 1.87 ± 0.42 μg/mg (*p* < 0.05), while no notable change was found in the BM group (Figure 6A). Similarly, the levels of valerate and isocaproic acid were markedly elevated in the FM group relative to the Control group (Figures 6E,H, *p* < 0.05). In contrast, the propionate level in the FM group significantly decreased compared with the Control group (Figure 6B, *p* < 0.05). Meanwhile, although the levels of butyrate, isobutyrate, and isovalerate increased, and hexanoate decreased, none of these changes reached statistical significance (*p* > 0.05) (Figures 6C,D,F,G).

### 3.5 Effects of fermented soymilk on metabolic functions of gut microbiota in mice

**FIGURE 7:**
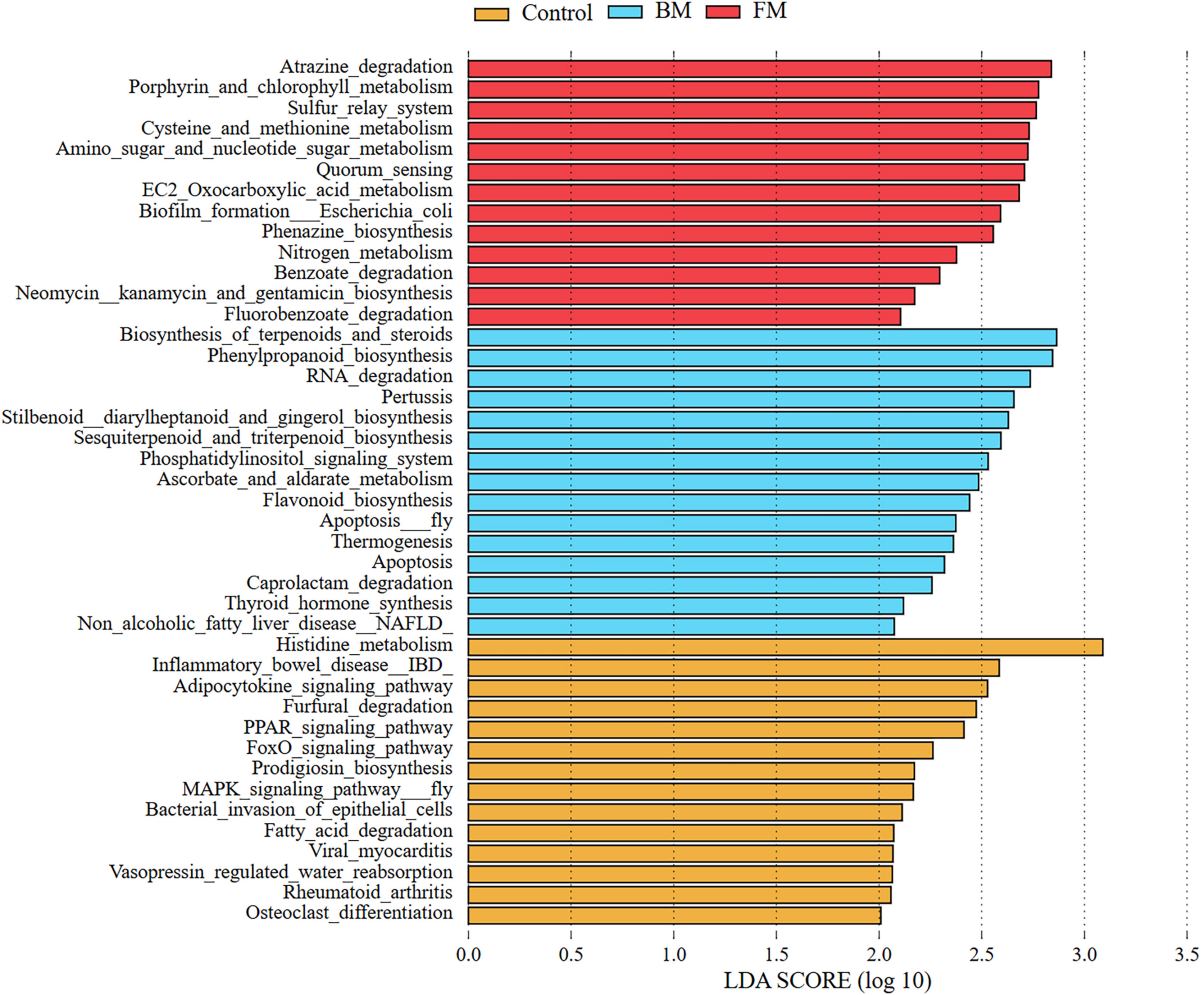
Linear discriminant analysis effect size (LEfSe) analysis identified differentially abundant KEGG pathways in level3 in the gut microbiome of mice among all groups (LDA score > 2.0, *p* < 0.05). Control: normal saline, BM: unfermented soymilk, FM: fermented soymilk.

Linear discriminant analysis effect size analysis demonstrated distinct functional shifts in the gut microbiota among all groups (Figure 7). Relative to the Control group, the BM group displayed enrichment in neural and immune-related pathways, whereas the FM group enriched 13 Kyoto Encyclopedia of Genes and Genomes (KEGG) pathways at level 3. Among these, pathways related to carbohydrate and amino acid metabolism, such as Amino sugar and nucleotide sugar metabolism, Cysteine and methionine metabolism, and 2-Oxocarboxylic acid metabolism, were significantly enriched, suggesting an enhanced capacity of the gut microbiota to metabolize carbohydrate and amino acid substrates following fermented soymilk intervention. Regarding xenobiotic degradation and plant-derived compound metabolism, Atrazine degradation, Benzoate degradation, and Fluorobenzoate degradation were significantly enriched, indicating an increased ability of the microbiota to degrade aromatic compounds. In addition, pathways related to microbial interaction (quorum sensing, biofilm formation) and secondary metabolism were enriched, indicating enhanced microbial communication and metabolic activity. Pathways involved in cofactor biosynthesis and elemental metabolism further supported an overall increase in microbial functional potential.

## 4 Discussion

Exercise-induced fatigue is defined as the failure of the body to maintain a specific exercise intensity (Lu et al., 2023). The forced swimming test with added weight represents a well-validated experimental protocol, applicable both to evaluating physical stamina and fatigue levels in laboratory animals and to detecting the anti-fatigue properties of test substances (Fang et al., 2022). In our study, the FM group showed a significantly improved exhaustive swimming time, indicating that continuous supplementation with fermented soymilk for 30 days significantly improved exercise endurance in mice, whereas no significant difference was found between the BM group and the Control group. During fermentation by kefir grains, soymilk can generate small peptides, more bioavailable aglycone-type soy isoflavones, and other bioactive microbial metabolites (Baú et al., 2015; Hasan et al., 2023). Consequently, fermented soymilk generally exhibits stronger biological activities than unfermented soymilk, including antioxidant, anti-inflammatory, lipid metabolism-regulating, and gut homeostasis-maintaining effects (Cai et al., 2021). Fang et al. (2022) also found that soybean protein peptides fermented by *Lactobacillus acidophilus* prolonged the exhaustive swimming time of mice.

Certain biochemical parameters measured after swimming can reflect the degree of fatigue in mice (Fang et al., 2022). BLA, an anaerobic sugar metabolite, accumulates during exercise, suppresses energy metabolism, and thus promotes fatigue via decreased muscle endurance (Bai, 2015). LDH serves as a key glycolytic enzyme and a common indicator of muscle damage (Yin et al., 2023). In our study, the FM group exhibited lower BLA levels than the Control and BM groups, however, this difference was not statistically significant. In contrast, LDH level in the FM group was significantly reduced, suggesting that fermented soymilk may alleviate exercise-related tissue stress and reduce muscle membrane damage. Furthermore, BUN level, which reflects protein catabolism, was also significantly decreased, indicating that fermented soymilk may reduce the breakdown of body protein during exercise and help maintain metabolic balance (Lu et al., 2023). The absence of a significant difference in CK levels among the groups may be attributed to the relatively mild degree of muscle injury induced by the current exercise model. Collectively, the changes in LDH and BUN levels in the fermented soymilk group indicated that fermented soymilk alleviated metabolic burden and enhanced the body’s resistance to exercise-induced physiological stress.

Oxidative stress is a key factor in fatigue development. Exercise induces excessive free radical production and disrupts cellular metabolism, thereby promoting fatigue (Lu et al., 2023). Free radical-mediated lipid peroxidation represents a major pathway of cell injury, directly damaging the cell membrane and being implicated in various diseases (Cheeseman, 1993). MDA, a product of lipid peroxidation generated upon oxygen free radical attack, serves as an indirect marker of cellular damage (Cand and Verdetti, 1989). In the present study, fermented soymilk significantly increased CAT activity and decreased MDA levels, indicating enhanced antioxidant defense and reduced lipid peroxidation. These results showed that fermented soymilk improves the ability of mice to resist exercise-induced oxidative stress.

The gut microbiota greatly impacts host metabolism (Clark and Mach, 2016), and studies have consistently demonstrated that it affects health by directly participating in or regulating various metabolic processes, often reflected by altered levels of microbiota-derived SCFAs (Luo et al., 2022; Lai et al., 2023). SCFAs are essential for health maintenance and serve as critical functional mediators connecting the gut microbiota to host physiology and pathology (Gomes et al., 2018). The gut microbiota remodeling induced by fermented soymilk was more pronounced than that induced by unfermented soymilk. At the phylum level, the abundance of Bacillota in the FM group was significantly increased. Bacillota are widely recognized as the major contributors to gut SCFA production and the principal decomposers of indigestible polysaccharides (Do et al., 2021). The FM group showed enrichment of various genera associated with anaerobic fermentation and the generation of SCFAs at the genus level, including *Enterocloster*, *Dysosmobacter*, *Lachnoclostridium*, *Mediterraneibacter*, *Blautia*, *Acutalibacter*, *Roseburia*, and *Faecalibacterium*, all of which belong to the phylum Bacillota. *Blautia* can ferment substrates such as glucose, sucrose, and fructose to produce acetate (Liang et al., 2025). The significant increase in fecal acetate content in the FM group further validated the alterations in microbial composition. As the most prevalent SCFA, acetate contributes significantly to host energy metabolism, immune regulation, and inter-organ signaling (Zheng et al., 2021). Together with *Faecalibacterium*, *Roseburia* is a key butyrate-producing genus in the gut that exhibits anti-inflammatory activity, preserves intestinal barrier permeability, protects the digestive system from enteric pathogens, and serves as a probiotic marker of gut health, making it a focal genus in future probiotic research (Qi et al., 2024). Recent research indicates that *Dysosmobacter*, which produces butyrate through a distinctive inositol-butyrate pathway, is closely linked to enhanced metabolic and hepatic health, positioning it as a next-generation probiotic candidate (Lee et al., 2016). *Lachnoclostridium* is also a major producer of butyrate (Ji et al., 2025). Notably, although the FM group was enriched in butyrate-producing genera, fecal butyrate content did not increase significantly. This discrepancy may be explained by the unique metabolic characteristics of butyrate in the host. Butyrate is the preferred energy substrate for colonic epithelial cells, with approximately 70%–80% of produced butyrate being rapidly absorbed and utilized via mitochondrial β-oxidation to meet the energetic demands of colonocytes (Gasaly et al., 2021; Guilloteau et al., 2010). Once absorbed, butyrate undergoes rapid metabolism in the gut wall and liver, resulting in undetectable levels in peripheral blood (Pravda, 2022). Consequently, fecal butyrate levels represent only the fraction that escapes host absorption and do not fully reflect the actual production level within the gut lumen. Moreover, mounting evidence suggests that fecal SCFAs levels are the integrated outcome of microbial production, host absorption, and eventual excretion (Hays et al., 2024; Nakatani et al., 2018). Therefore, the lack of correlation between butyrate-producing bacterial abundance and fecal butyrate content does not necessarily indicate reduced butyrate production, rather, it may reflect efficient host utilization. This interpretation is further supported by Park et al. (2024) that butyrate oxidation by colonocytes is essential for promoting the growth of butyrate-producing bacteria, establishing a host-microbiome mutualistic relationship based on butyrate metabolism.

It is noteworthy that fermented soymilk also significantly elevated the contents of valerate and isocaproic acid in the gut of mice compared with the Control group. Luo Z. et al. (2023) reported that valeric acid treatment (100 mg/kg/day, 5 weeks) significantly increased gastrocnemius muscle mass and myofiber cross-sectional area, up-regulated MyHC IIb expression, enhanced glycolytic enzyme levels (HK2, PFK1, PKM2), activated the Akt/mTOR pathway, and improved grip strength in mice. Changes in fecal isocaproic acid abundance are closely associated with the host’s intestinal health status. A clinical study involving 183 middle-aged and elderly men found that isocaproic acid predominated in the fecal branched-chain short-chain fatty acid (BCFA) profile of the healthy control group (Ratajczak et al., 2021). This suggests that a high abundance of isocaproic acid may be related to normal intestinal barrier function and homeostasis. In the present study, fecal isocaproic acid levels in the FM group were significantly elevated, accompanied by a marked reduction in the abundance of potentially pathogenic genera such as *Helicobacter* and *Escherichia*. These findings suggest that fermented soymilk may promote the metabolic activity of isocaproic acid-producing bacteria, including *Clostridium* species, by suppressing opportunistic pathogens and optimizing the gut microecology.

Furthermore, functional prediction revealed that the FM group showed significant enrichment of pathways involved in amino acid metabolism, carbohydrate metabolism, xenobiotic degradation, quorum sensing, and biofilm formation. These changes indicate that fermented soymilk not only altered microbial composition but also enhanced the functional potential of the gut microbiota. In particular, the enrichment of pathways related to carbohydrate and amino acid utilization may reflect an enhanced capacity of the microbiota to convert dietary substrates into metabolites relevant to host energy homeostasis.

## 5 Conclusion

**FIGURE 8:**
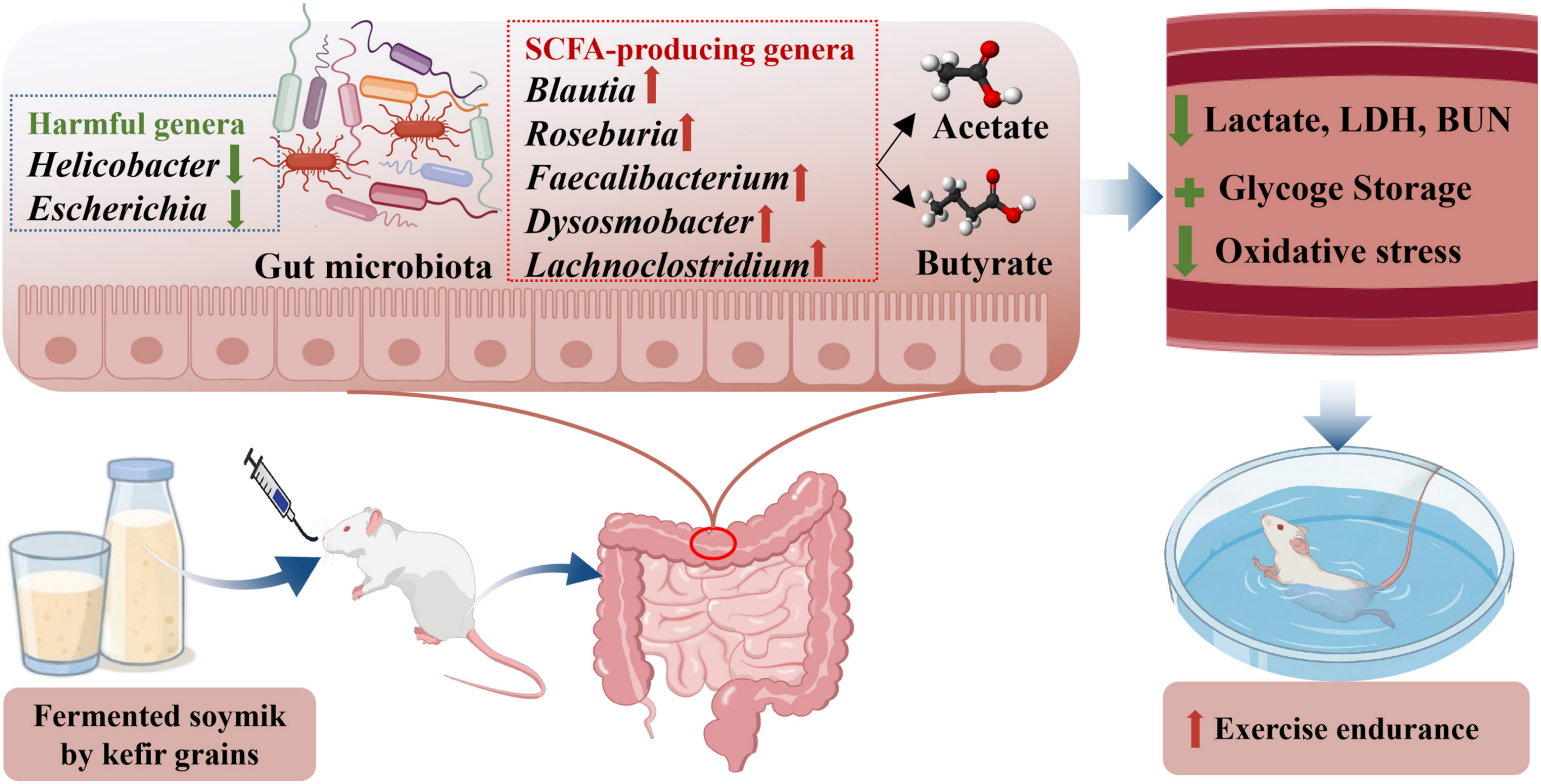
Proposed mechanisms underlying the effects of fermented soymilk on attenuating exercise-induced fatigue.

This study revealed that fermented soymilk supplementation significantly enhanced exercise endurance and reduced fatigue in mice. The findings indicated that fermented soymilk regulated the abundance and proportion of the gut microbiota. Specifically, it enriched SCFA-producing bacteria, such as *Blautia*, *Faecalibacterium*, *Roseburia*, *Dysosmobacter*, and *Lachnoclostridium*, while suppressing opportunistic pathogens like *Helicobacter* and *Escherichia*. Moreover, it improved the capacity for dietary substrate conversion, promoted SCFAs (acetate and butyrate) synthesis, facilitated hepatic and muscular glycogen synthesis, enhanced energy reserves, strengthened intestinal barrier function, alleviated oxidative stress, and thereby ameliorated exercise-induced fatigue (Figure 8).

## Data Availability

The datasets presented in this study can be found in online repositories. The names of the repository/repositories and accession number(s) can be found below: https://www.ncbi.nlm.nih.gov/bioproject/1466123, PRJNA1466123.
